# A likelihood‐based particle imaging filter using prior information

**DOI:** 10.1002/mp.16258

**Published:** 2023-02-18

**Authors:** Ryan Fullarton, Lennart Volz, Nikolaos Dikaios, Reinhard Schulte, Gary Royle, Philip M. Evans, Joao Seco, Charles‐Antoine Collins‐Fekete

**Affiliations:** ^1^ Department of Medical Physics and Biomedical Engineering University College London London UK; ^2^ Department of Biomedical Physics in Radiation Oncology Deutsches Krebsforschungszentrum (DKFZ) Heidelberg Germany; ^3^ Department of Physics and Astronomy Heidelberg University Heidelberg Germany; ^4^ GSI Helmholtz Centre for Heavy Ion Research GmbH Darmstadt Germany; ^5^ Centre for Vision Speech and Signal Processing University of Surrey Guildford UK; ^6^ Mathematics Research Center Academy of Athens Athens Greece; ^7^ Department of Basic Sciences Division of Biomedical Engineering Sciences Loma Linda University Loma Linda California USA; ^8^ Chemical, Medical and Environmental Science National Physical Laboratory Teddington UK

**Keywords:** bespoke filter, helium CT, ion imaging, noise reduction, proton CT

## Abstract

**Background:**

Particle imaging can increase precision in proton and ion therapy. Interactions with nuclei in the imaged object increase image noise and reduce image quality, especially for multinucleon ions that can fragment, such as helium.

**Purpose:**

This work proposes a particle imaging filter, referred to as the Prior Filter, based on using prior information in the form of an estimated relative stopping power (RSP) map and the principles of electromagnetic interaction, to identify particles that have undergone nuclear interaction. The particles identified as having undergone nuclear interactions are then excluded from the image reconstruction, reducing the image noise.

**Methods:**

The Prior Filter uses Fermi–Eyges scattering and Tschalär straggling theories to determine the likelihood that a particle only interacts electromagnetically. A threshold is then set to reject those particles with a low likelihood. The filter was evaluated and compared with a filter that estimates this likelihood based on the measured distribution of energy and scattering angle within pixels, commonly implemented as the 3σ filter. Reconstructed radiographs from simulated data of a 20‐cm water cylinder and an anthropomorphic chest phantom were generated with both protons and helium ions to assess the effect of the filters on noise reduction. The simulation also allowed assessment of secondary particle removal through the particle histories. Experimental data were acquired of the Catphan CTP 404 Sensitometry phantom using the U.S. proton CT (pCT) collaboration prototype scanner. The proton and helium images were filtered with both the prior filtering method and a state‐of‐the‐art method including an implementation of the 3σ filter. For both cases, a dE‐E telescope filter, designed for this type of detector, was also applied.

**Results:**

The proton radiographs showed a small reduction in noise (1 mm of water‐equivalent thickness [WET]) but a larger reduction in helium radiographs (up to 5–6 mm of WET) due to better secondary filtering. The proton and helium CT images reflected this, with similar noise at the center of the phantom (0.02 RSP) for the proton images and an RSP noise of 0.03 for the proposed filter and 0.06 for the 3σ filter in the helium images. Images reconstructed from data with a dose reduction, up to a factor of 9, maintained a lower noise level using the Prior Filter over the state‐of‐the‐art filtering method.

**Conclusions:**

The proposed filter results in images with equal or reduced noise compared to those that have undergone a filtering method typical of current particle imaging studies. This work also demonstrates that the proposed filter maintains better performance against the state of the art with up to a nine‐fold dose reduction.

## 1 INTRODUCTION

Ion computed tomography is an imaging method that uses charged particles (often protons or helium ions) to create a 3D map of material parameters (stopping power, scattering power, nuclear attenuation, straggling in reference to water) from an object.^1^ It presents several advantageous properties compared to conventional X‐ray CT imaging, among which the three most important are (1) a lower imaging dose,^2^, ^3^ (2) low sensitivity to metal or cupping artifacts usually present in X‐ray CT and (3) the possibility to reconstruct multiple images from a single‐acquisition.^1^ It has been proposed for a variety of clinical applications, such as particle therapy treatment planning,^4^ multimodality imaging,^5^ dynamic motion tracking,^6^, ^7^ and patient positioning.

Ion tomography image formation is different from that of x‐ray tomography, relying on charged particles that interact with nuclei through the Coulomb scattering processes and lose energy through interactions with atomic electrons, to form the reconstructed image. The nuclei scattering process induces a blurring in the reconstructed image, which depends on the properties of the chosen ion.^8^ Therefore, particle imaging is commonly used as a single‐event modality, to facilitate path reconstruction and improve image quality. Since every recorded particle contributes to the final image, this method of reconstruction also minimizes the required dose to produce high‐quality images. Still, some processes undergone by the charged particles through the medium induce noise in the reconstructed image, such as large‐angle Coulomb scattering or nuclear interactions.

To maximize image quality in particle imaging for its promising applications, bespoke filters must be developed that account for the different processes and the single‐event nature of this modality. Commonly, data filtering in particle imaging is based on the mean and standard deviation of the distribution of the measured energy loss and angular displacement of particles that are binned based on their measured positions. This follows the reasoning that the central part of both energy straggling and angular distribution approximate Gaussian distributions. Particles that have undergone large‐angle scattering or nuclear fragmentation are not likely to be well represented by this distribution. This allows particle filtering based on their distance from the distribution mean by applying a strict cutoff ($\sigma_{t})$, such as a multiple of the distribution's standard deviation, beyond which they are not included in image reconstruction.

A filter of this kind was initially presented by Schulte et al.,^3^ where a threshold of $\sigma_{t}=3\sigma$ was explored and later again investigated by Schulte et al.^9^ with a threshold of $\sigma_{t}=2\sigma$, but in theory, any cut off value can be used. It may, however, perform poorly in a heterogeneous medium, where the distribution of all particles is a poor surrogate for the state of an individual particle.^10^ Despite being the filter approach used most often in literature, there is no agreed‐upon standard of how to perform the 3σ filtering. For example, the binning of the particles to form the distributions underlying the filtering can be performed based on the particle's position on the front or rear tracker, or any position in between, using an estimate of their path. The position where the particles are binned has a considerable effect on the point‐spread function for features located at different depths in the object,^11^ and, therefore, also the computation of the pixel‐wise standard deviation underlying the filtering. Finally, when the pixel‐wise distributions are formed, the standard deviation can be computed in different ways. Computing the standard deviation directly from the distribution variance is highly sensitive to the tails of the non‐Gaussian distributions of WEPL^12^ and angular deviation. This issue is amplified for experimental pCT setups, due to additional detector noise. In literature, rough cuts to the distribution are, therefore, sometimes applied prior to the variance calculation.^13^ Alternatively, the standard deviation can be computed from a specific fall‐off point of the distribution, for example, the FWHM. However, while this is more robust to distribution tails,^14^ it is nonideal when there is more than one peak in the distribution. For this work, this type of filter will be referred to as the “sigma filter,” to reflect the fact that it works by making predictions based on the standard deviation of distributions measured at positions in the projection data, when not referring to the filter implemented with a specific threshold.

To improve on the sigma‐based filtering, Volz et al. have introduced, first for helium ions^15^ and later for protons,^13^ particle discrimination using a longitudinally segmented calorimeter as a dE‐E telescope. They have demonstrated that it is possible to discriminate the incident particle charge and mass and consequently identify it for acceptance/rejection. This method has shown highly promising results in the reconstruction, reducing the noise drastically in the image. However, this method relies on projectile mass/charge variation and is not able to discriminate (1) nuclear elastic events, (2) nuclear inelastic events where the projectile integrity is conserved as well as (3) large angle Coulomb scattering events. Therefore, further filtering is required to achieve optimal image quality.

This work presents a novel, physics‐based, filter for particle imaging that (1) models the interactions of charged ions based on electromagnetic processes and (2) calculates a likelihood of the difference between detected and predicted characteristics, for individual particles, based on the model. Similarly, to the sigma filter, this filter uses Gaussian distributions to try and identify particles that have undergone nuclear interactions or large angle scattering. However, rather than comparing each particle to others in its detected bin, the proposed filter bases its likelihood estimate on how well the measured particle position, deflection, and energy loss agree with predicted values obtained through a model of electromagnetic interactions and a prior estimate of the imaged object. The aim of this work is to further reduce the effects of secondary particles and large‐angle scattering on image quality, and enable low‐dose high‐quality tomographic particle imaging.

## 2 MATERIALS AND METHODS

The filter as implemented in this work modeled the electromagnetic interactions of primary particles to identify particles that had undergone nuclear interactions and exclude them from the reconstruction. The model used prior information, namely an estimated relative stopping power (RSP) map, to predict the measured exit energy and position of each individual particle used in image acquisition, and estimate the likelihood it is a primary particle, as described in Section 2.1. To evaluate the filter, simulated and experimental particle images (proton and helium ions) were reconstructed, after being filtered using the proposed filter (referred to as prior filtering) and compared to radiographs being reconstructed after filtering with the state‐of‐the‐art sigma filter. Simulated radiographs of a water cylinder and the anthropomorphic XCAT phantom^16^ (Section 2.2) were used to assess intrapixel noise and the efficiency of the two filters to remove secondary particles. For the prior information required to filter the simulated data, an analytical description of the water cylinder with an RSP of 1.0 was used and the voxelized phantom itself was used for the XCAT phantom. The experimental data (Section 2.3) include tomographic reconstructions of the Catphan CTP404 sensitometry module, using the distance‐driven binning (DDB) reconstruction algorithm^17^ (Section 2.4), to generate back projected tomographs of the image noise for filter evaluation. The prior information was obtained by first reconstructing a tomograph using state‐of‐the‐art filters. A full description of how each filter is applied and the processing order is given in Section 2.5.

### 2.1 Primary model: Electromagnetic interactions

**FIGURE 1:**
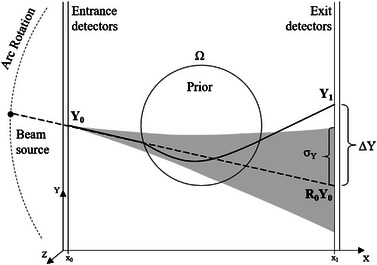
A visual representation of an ion computed tomography apparatus, and parameters used in the primary model, used in this study. Shown are the pair of double‐layered detection planes, the beam source rotating around the phantom, the entrance (*Y*_0_), the measured (*Y*_1_) and predicted ($Y_{0}R_{0}$) parameter vectors, the calculated standard deviation in the *Y* direction ($\sigma_{y}$) as well as the prior information, a previous reconstruction of the phantom with state of the art filtering for this study, (Ω).

A visualization of the ion tomography apparatus and the parameters used for the model of the interaction of primary particles is shown in Figure 1. The primary particles will refer to those that have undergone small‐angle electromagnetic interactions only and all others will be referred to as secondaries. The model for these particles is split into the Coulomb scattering and energy loss processes, respectively, making use of Fermi–Eyges and Bethe–Bloch/Vavilov theories.^18^, ^19^ It is defined as a multivariate normal distribution centered on the difference between the measurements and their expectations. The predicted position and direction are projected as a straight line from the front tracker position and direction measurement (dashed line in Figure 1). Their standard deviation is obtained from the Fermi–Eyges theory^20^ (shaded region in Figure 1). The expected energy out is acquired by superposing a path estimate^21^ (solid line in Figure 1) to a prior image of the phantom (Ω) combined with the Bethe–Bloch equation. The standard deviation of the energy loss is obtained from the Tschalär theory.^12^ The primary model can, therefore, be expressed as

(1) $$L_{prim.}=N_{2}\left(\Delta Y,\Sigma \right)\cdot N_{2}\left(\Delta Z,\Sigma \right)\cdot N\left(\Delta E,\sigma_{E}\right)$$

In Equation (1), the operator N represents the single variate normal distribution and $N_{2}$ the bivariate normal distribution. Let us define a parameter vector as *Y* = $\left(y,\theta_{y}\right)$ where *y* is the position, and $\theta_{y}$ is the component of the particle's direction along the *y*‐axis. *Y*_0_ represents this position as measured by the front tracker, at $x=x_{0}$, and *Y*_1_ the position as measured by the rear tracker at $x=x_{1}$. ΔY and ΔZ represent the difference between the exit measured parameter vector and the predicted one, respectively, in the lateral and longitudinal axis defined in Section 2.1.1. Σ is the co‐variance matrix, which is identical in both dimensions and is defined in Section 2.1.1. ΔE is the difference between the measured and predicted energy loss and $\sigma_{E}$ represents the energy loss standard deviation as described in Section 2.1.2. Using this model to calculate a likelihood for each particle, given predicted exit vectors and energy loss, particles that fall below a likelihood threshold can be rejected as likely to have undergone processes not included in this model, that is, large‐angle scattering or nuclear interactions. This threshold can be placed on the overall likelihood or on the individual predictions (i.e., $N<P_{t}$ or $L_{prim.}<P_{t}^{3}$ where $P_{t}$ is the threshold value). For clarity, the threshold values quoted later in this work will be the threshold applied to each of the individual predictions. Due to the use of normal distributions to calculate the likelihood, the Prior Filter threshold value ($P_{t}$) could be expressed in terms of standard deviation, that is, 1σ would be $P_{t}=0.68$. To represent the filter as implemented and to avoid confusion with the sigma filter threshold ($\sigma_{t}$) the threshold values used in this work will be reported as a probability value between 0 and 1.

#### 2.1.1 Fermi–Eyges lateral and angular displacement model

The multiple Coulomb scattering is modeled using the Eyges solution^20^ to Fermi theory^22^ that models small angle scattering in a multivariate normal distribution. A particle can be deflected in any direction in two dimensions perpendicular to its path. Here, the scattering distribution will be defined for a single plane representing one of the two perpendicular dimensions. The orthogonal scattering distribution will be assumed to be predicted by the same rules. The scattering in the two perpendicular planes is uncorrelated and can thus be treated independently. The multivariate normal function can be defined as

(2) $$N_{2}\left(\Delta Y,\Sigma_{y}\right)=\frac{\exp\left(\frac{-1}{2}{\left(\Delta Y\right)}^{T}\Sigma^{-1}\left(\Delta Y\right)\right)}{\sqrt{{\left(2\pi \right)}^{2}\left|\Sigma \right|}}$$

where $N_{2}$ represents a bivariate normal distribution, ΔY is calculated from the parameter vector $Y_{1}=\left(y_{1},\theta_{y1}\right)$, the exit measurement, and $Y_{0}=\left(y_{0},\theta_{y0}\right)$, the entrance measurement.

(3) $$\Delta Y=Y_{1}-R_{0}Y_{0}$$

*R*_0_ is defined as the transvection matrix that projects the entrance measurement to the exit tracker, to form the predicted exit position, and is defined as

(4) $$R_{0}=\left(\begin{array}{cc} 1 & x_{1}-x_{0}\\ 0 & 1 \end{array}\right)$$

The covariance matrix is defined as

(5) $$\Sigma =\left(\begin{array}{cc} \sigma_{y}^{2} & \sigma_{y-\theta }^{2}\\ \sigma_{y-\theta }^{2} & \sigma_{\theta }^{2} \end{array}\right)$$

The moments of the distribution are defined with Highland's correction^23^ to the Eyges MCS theory^24^:

(6) $$\sigma_{\theta }^{2}={\left(1+\frac{1}{9}ln\left(\int_{x_{0}}^{x_{1}}\frac{dx}{X_{0}\left(x\right)}\right)\right)}^{2}\int_{0}^{x_{1}}{\left(\frac{z\cdot E_{0}}{pv(x)}\right)}^{2}\frac{dx}{X_{0}\left(x\right)}$$

(7) $$\sigma_{y-\theta }^{2}={\left(1+\frac{1}{9}ln\left(\int_{x_{0}}^{x_{1}}\frac{dx}{X_{0}\left(x\right)}\right)\right)}^{2}\int_{0}^{x_{1}}{\left(\frac{z\cdot E_{0}}{pv(x)}\right)}^{2}\frac{(x-x_{1})dx}{X_{0}\left(x\right)}$$

(8) $$\sigma_{y}^{2}={\left(1+\frac{1}{9}ln\left(\int_{x_{0}}^{x_{1}}\frac{dx}{X_{0}\left(x\right)}\right)\right)}^{2}\int_{0}^{x_{1}}{\left(\frac{z\cdot E_{0}}{pv(x)}\right)}^{2}\frac{{\left(x-x_{1}\right)}^{2}dx}{X_{0}\left(x\right)}$$

The term $X_{0}\left(x\right)$ represents the radiation length of the material at depth *x*, which was approximated to be water whenever inside the object (36.1 cm) and air outside the object (3.5 × 10^4^ cm). This assumption is not expected to greatly affect the path reconstruction, as this has been demonstrated to be an appropriate approximation in previous work.^25^ The empirical constant *E*_0_ =14.1 MeV was used as in Gottschalk et al.^26^ to best represent the scattering of ions crossing a medium. The term pv(x) represents the momentum and velocity as a function of the depth in the material, specific to the investigated ion. It can be described through the energy function of the particles throughout the medium. In this work, the prior image allows us to generate the pv(x) function and calculate the integral directly as

(9) $$pv\left(x\right)=\frac{E\left(x\right)+2m_{I}c^{2}}{E\left(x\right)+m_{I}c^{2}}E\left(x\right)$$

where E(x) is the energy of the ion at depth *x* and $m_{I}$ is the rest mass of the ion species used for imaging. Finally, *z* represents the charge of the particle for which the scattering power is calculated.

#### 2.1.2 Energy loss model and energy straggling

The calculation of the expected energy loss is done by constructing a maximum likelihood path (MLP) estimate **S**(*t*) through an initial reconstruction of the phantom, following the procedure shown in Collins‐Fekete et al.^25^ This reconstruction provides the prior‐knowledge RSP map. The predicted exit energy of each particle can be calculated following the continuous‐slowing‐down approximation as

(10) $$E_{pred.}=E_{init}-\int_{0}^{t}RSP\left(S\left(t\right)\right)SP(I_{w},E\left(S\left(t\right)\right)dt$$

The parameterization of the MLP, S(t), using a phenomenological adaptation of a cubic spline as proposed by Collins‐Fekete et al.^21^ was used combined with a convex hull to account for the air gap.^25^, ^27^ $I_{w}$ is the water mean excitation energy set at 75 eV for this work, SP($I_{w},E$) is the water stopping power at a given energy *E*, $E_{init}$ is the initial energy. RSP(S(t)) is the local RSP, which is taken from the prior‐knowledge RSP map along the path estimate of the particle.

The energy loss likelihood is defined by the difference between the expected exit energy (Equation 10) and the measured energy, with the modeled fluctuation around this value (Equation 11). For two protons following an identical path through a medium, the amount of energy loss is subject to two sources of fluctuations: The number of proton–electron collisions can fluctuate statistically, as well as the energy loss in each collision. The energy variation introduced by these fluctuations is called energy straggling. Energy straggling has been described thoroughly for thin absorbers by Vavilov^19^ and approximated for thick absorbers by Tschalär^12^:

(11) $$\sigma_{E}^{2}\left(E_{out}\right)=k_{1}^{2}\left(E_{out}\right)\int_{E_{0}}^{E_{out}}\frac{k_{2}\left(E_{out}\right)}{k_{1}^{3}\left(E_{out}\right)}dE$$

where $E_{out}$ is the energy of an ion exiting the phantom and *k*_1_ and *k*_2_ are defined as

(12) $$k_{1}=\frac{K}{\beta^{2}\left(E\right)}\left[\ln\left(\frac{2m_{e}c^{2}}{I(S(t))}\frac{\beta^{2}\left(E\right)}{1-\beta^{2}\left(E\right)}\right)-\beta^{2}\left(E\right)\right],\;k_{2}=\eta_{e}K\frac{1-1/2\beta^{2}\left(E\right)}{1-\beta^{2}\left(E\right)}$$

In this equation, *c* is the speed of light, β is the particle velocity relative to the speed of light, $\eta_{e}$ is the ratio of the electron density of the medium to the electron density of water, $m_{e}$ is the relativistic electron rest mass, I(S(t)) is the local medium mean excitation energy, which in this work is approximated as water (75 eV), and the constant *K*= 170 MeV/cm combines various fixed physical parameters. The likelihood model for the energy loss of primary particle is defined as

(13) $$N\left(\Delta E,\sigma_{E}\right)=\frac{1}{\sqrt{2\pi \sigma_{E}^{2}}}\exp\left(\frac{-\Delta E^{2}}{2\sigma_{E}^{2}}\right)$$

where ΔE is defined as the difference between the measured exit energy ($E_{out}$) and the predicted exit energy ($E_{pred}$).

### 2.2 Geant4 Monte Carlo simulations

For evaluation, radiograph acquisitions were simulated using the Geant4 MC code version 10.06.p03.^28^ 200 MeV/u ions (*n* = 10^7^) in a 30 cm × 30 cm homogeneous field were simulated through a cylinder of water with a diameter of 20 cm and the XCAT anthropomorphic body phantom.^16^ The phantoms were located at the center of the two imaging planes, with a 5 cm air gap between the detectors and the phantom. The detectors in these simulations were treated as ideal planes; one upstream and one downstream from the imaged object. The primary beam was distributed evenly along the phantom lateral side with no initial angular deviation, neglecting any beam‐line effects. Every particle was recorded with the process that generated it, the water‐equivalent path length (WEPL), converted from the recorded exit energy, and both its initial and final position and direction.

The standard physical processes included energy loss and straggling, following Bethe–Bloch theory, multiple Coulomb scattering based on Lewis theory^29^ using the Urban model,^30^ a definition of parameterized interactions with nuclei and electrons and elastic/inelastic ion interactions from Geant4 ion dedicated packages.^31^ The following physics lists were enabled: the standard electromagnetic option 3 for higher accuracy of electrons, ions, and ion tracking without a magnetic field, the ions elastic model (G4HadronElasticPhysics) and a mix of the QMD (G4IonQMDPhysics) and the Bertini/binary cascade (G4HadronInelasticQBBC) ion models both for elastic and inelastic collisions. Electrons, neutrons, and gamma particles were removed from the analysis as they would be easily removed by existing filters, allowing the analysis to better reflect the more difficult cases. Finally, one major distinction was introduced within the Geant4 simulation; particles identified with a hadron elastic scattering were classified as secondary particles.

### 2.3 Experimental data

**FIGURE 2:**
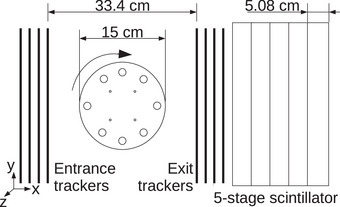
Schematic of the experimental setup for the acquisition proton and helium tomographs of the CTP404 module using the U.S. pCT collaboration scanner. pCT, proton CT.

Experimental proton and helium CT data, to test the filter performance, were available from a recent study conducted with the US pCT collaboration prototype particle CT scanner^32^ at the Heidelberg Ion‐Beam Therapy Center (HIT). A schematic of the experimental acquisition is shown in Figure 2. The prototype scanner comprised of eight silicon strip tracking planes (0.228 mm strip pitch), arranged into a front and a rear tracker, measuring the position and direction of each particle. The trackers have an aperture of 350 × 90 mm^2^, and the distance between the innermost tracker layers was 334 mm. A five‐stage scintillator placed behind the rear tracker was used to measure the residual energy/range of each particle after passing the object.

The five polystyrene scintillator blocks, each 50.8 mm thick in the beam direction and are read out by a single photo‐multiplier, are arranged sequentially such that the thickness of each stage crossed completely by a particle directly contributes to the measurement of its residual range. Only in the stage, where the particle stopped, is the measured energy deposit relevant, relaxing precision requirements of the detector readout and improving overall image noise.^33^

The prototype featured a remote‐controlled rotating platform that enabled the acquisition of full CT scans at horizontal beam lines, either in stepped or continuous acquisition mode. For this work, pCT and HeCTs scans of a Catphan (the Phantom Laboratory, Salem, NY, USA) CTP404 sensitometry module were acquired in stepped scan mode from 360 projections separated at 1° steps. The phantom was a 150 mm diameter, 20 mm tall epoxy cylinder that contained six different cylindrical plastic inserts. RSP reference values for each of the inserts were available from Giacometti et al.^34^

The initial energy was 200.11 MeV for protons, and 200.38 MeV/u for helium ions. Each projection consisted of approximately 1.4× 10^6^ irradiated particles at ∼700‐kHz particle rate for helium ions, and 3.1 × 10^6^ particles irradiated at ∼1.35‐MHz particle rate for protons. Irradiation fields of 200× 50 mm^2^ were generated employing the HIT raster scanning technique. Thereby, the beam FWHM was 10.2 mm for helium ions and 12.8 mm for protons. The lateral spacing between spots was 3 mm. The differences in particle rates were due to vendor‐set sensitivity limits in the beam monitoring systems regulating the raster scanning. The high proton rate resulted in reduced dose efficiency for pCT compared to other published literature on pCT with the scanner, due to both detector dead time and event pile‐up.

Before image acquisition, the five‐stage energy/range detector was calibrated to water‐equivalent thickness (WET) using a dedicated setup of known geometry and RSP. The procedure is detailed in the recent technical report by Schultze et al.,^35^ and results in individual energy‐to‐WET calibration curves for each of the five scintillator stages. When processing each set of projection data, first, an online re‐calibration of the photo‐multiplier gain is performed using particles that missed the phantom to ensure the highest accuracy of the energy measurement. Then, the energy output from the stage where a particular event stopped was used to assign it a WET value from the calibration curve.

All experimental data made use of the dE‐E telescope filter as described by Volz et al.,^13^, ^15^ which utilizes the multiple segments to remove secondary particles, providing the best impression of image quality achievable. However, for completeness, the CT reconstructions without use of the dE‐E filter are included in Appendix A. The simulated data does not use this filter due to the use of ideal plane detectors in the simulation.

### 2.4 CT and tomographic noise reconstruction

For tomographic reconstruction, the DDB method of Rit et al.^17^ was used. This reconstruction technique was chosen over nonlinear reconstruction methods to allow back projection of the pixel noise and better evaluate the Prior Filter performance. The reconstruction grid used 320 bins in both the *y* and *z* direction with a field of view covering 200 mm (0.625 mm in‐plane pixel size) with 1 mm slices in the *x* direction. The implementation of this reconstruction binned the WEPL values of each particle along the MLP parameterized using the cubic spline method of Collins‐Fekete et al.^21^ for each projection, before convolving it with the Ram‐Lak filter.^36^ All the projections were then summed to generate the tomograph. To reconstruct the tomographic noise, the binning was conducted using the same method, but the standard error of the WET calculated using all the particles for each bin of a projection ($\sigma_{\theta ,\mathrm{WET}}^{2}\left(y\right)$) was back‐projected convolved with the squared Ram‐Lak filter function^37^, ^38^

(14) $$\sigma^{2}\left(x,y\right)=B\left\{\frac{\sigma_{\theta ,\mathrm{WET}}^{2}\left(y\right)}{N_{D}\left(y\right)}⊛h^{2}\left(y\right)\right\}.$$

where B represents the back‐projection operator, *h* represents the filtering function, and $\sigma_{\theta ,WET}$ represents the standard error in each bin (expressed in WET) for a defined position/projection angle. From the variance, the intrapixel noise can be calculated by taking the square root. The sigma filter and Prior Filter were applied before reconstructing with the DDB algorithm. The reconstruction as implemented here assumes a parallel beam, due to the distance from the scanning magnets to the center of the phantom (6.5 m and 7.2 m) being much larger than the diameter of the phantom. Also, only the central slice was used in the analysis to minimize the cone beam effects in the vertical direction.

### 2.5 Filtering and comparison metrics

#### 2.5.1 State‐of‐the‐art filter steps

Prior to applying any of the filters under investigation, all cases were filtered with hard cuts at 0 and 260 mm WEPL to remove physically nonmeaningful values from the dataset. The state‐of‐the‐art filtering included the sigma filter (investigated up to thresholds ($\sigma_{t}$) of 10σ) with radiographs and tomographs presented using the 3σ threshold suggested by Schulte et al.^9^ and used for the DROP‐TVS reconstruction.^39^ In the rest of the text, when referring to the sigma filter being used with a threshold ($\sigma_{t}$) of 3σ this filter will be named as the 3σ filter and when no specific threshold is being applied it will continue to be referred to as the sigma filter. The dE‐E telescope filter was also applied to the state‐of‐the‐art datasets to identify and remove ions resulting from fragmentation^13^, ^15^ in the experimental data. For the sigma‐based filtering, each projection was binned into a grid with bins of 1 mm × 1 mm based on their measured exit positions. The mean and standard deviation of both WEPL and scattering angle ($\theta_{s}$) were calculated for each bin.

(15) $$\theta_{s}=\sqrt{{\left(\theta_{y,0}-\theta_{y,1}\right)}^{2}+{\left(\theta_{z,0}-\theta_{z,1}\right)}^{2}}$$

To avoid the effects of the distribution tails on the calculation of parameters in the sigma filter, for each bin, values were included in the calculation of mean and standard deviation if they fell within 0.7 × the bin mode up to 1.3 × the bin mode within their bin.^13^ For the data cuts, any particles with a WEPL or scattering angle that lies outside the threshold ($\sigma_{t}$) for its bin were removed prior to reconstruction. The filtered data were then used to reconstruct 2D radiographs and 3D tomography images. As discussed in Section 1, there are inconsistent methods used in implementing the sigma filter in the literature. Although this one has been chosen for a full investigation, due to its common usage, a comparison to other implementations has been included in Appendix B.

#### 2.5.2 Prior filter steps

As with the state‐of‐the‐art filtering, hard cuts at 0 and 260 mm WEPL were made to remove unrealistic values before applying any other filters. In order to use the proposed filter, prior information (i.e., an estimated RSP map of the object) was required. For the simulated water cylinder acquisition, the prior information was an analytically described cylinder with the same geometry as the simulation and an RSP of 1.0. For the XCAT phantom, the voxelised known RSP, as used in the simulation, was used to create a 3D phantom for use as the prior information. In effect, the prior information for the simulated data is an example of perfect or ideal prior information.

**FIGURE 3:**
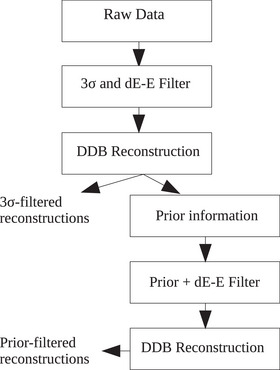
A flow chart showing the steps of generating the state‐of‐the‐art 3σ reconstructions and how these reconstructions are used as prior information for filtering using the methods proposed here. This process follows the steps of processing that were applied to the experimental data in this study.

For the experimental data, no ideal prior information was available, so a different method is required, which is shown in Figure 3 and described here. An initial image reconstruction, with the state‐of‐the‐art filters applied, served as the prior reconstruction to provide the RSP map for use in the Prior Filter. In effect, the experimental data followed the process that we envision for potential future adoption of this method in particle imaging experiments or in clinics. First, the acquired data were reconstructed with both the 3σ threshold and dE‐E telescope filter applied as in the state‐of‐the‐art reconstructions. Second, as an additional step, this initial reconstruction provides the RSP map for the Prior Filter. Once the Prior Filter has been applied, a second tomographic reconstruction was done to provide the Prior Filtered tomographs.

The estimates of standard deviation in the model depend on the thickness of the material traversed, which can result in unreasonably narrow uncertainty envelopes for particles that cross only a small section of the phantom. Additionally, the model described in Section 2.1 assumes perfect measurement of each parameter. To take these factors into account, a minimum value of 3 mm in positional prediction was enforced when the standard deviation predicted by the model was lower than 3 mm. For ease of calculation, the energy loss predictions were parameterized by the WEPL of the particle. In practical terms, this allowed the predicted value to be determined by integrating the RSP in the prior reconstruction along the particle's path. As with the positional predictions, a minimum standard deviation of 3 mm of WEPL was applied to particles with a lower standard deviation predicted by the model. Because the sigma filter uses the measured data to determine its parameters, it would not return such an unrealistic value. The threshold for each of the Prior Filter parameters was set at 0.6 for proton data and 0.4 for helium data. The reason two different values were selected will become clear in Section 3.1.2, where we investigate the filter performance as a function of the threshold values.

#### 2.5.3 Filter comparison

The filter performance was compared in terms of mean noise profiles. These were produced by averaging the intrapixel noise along the vertical direction (*y*‐direction in Figures 1 and 2) of the radiographs, reconstructed on the front tracker plane, to create a single horizontal profile of the noise from the filtered datasets. Additionally, a tomographic reconstruction of the experimental data is also presented using the DDB method^17^ along with the back‐projected noise for the sensitometry phantom as specified in Section 2.4.

We also evaluated the specificity and sensitivity of both Prior and Sigma filters under various thresholds following:

(16) $$Sensitivity=\frac{True\;Positives}{True\;Positives+False\;Negatives}$$

(17) $$Specificity=\frac{True\;Negatives}{True\;Negatives+False\;Positives}$$

where true positives are secondaries correctly identified as secondaries, false negatives are secondaries incorrectly identified as primaries, true negatives are primaries correctly identified as primaries and false positives are primaries incorrectly identified as secondaries. This was possible using the known interaction histories of the simulated data, where any particles that did not exclusively follow the modeled electromagnetic interactions were considered secondaries. Although events such as inelastic collisions are easily identifiable in the Monte Carlo histories, it would be difficult to define and differentiate particles that have undergone large angle Coulomb scattering with electrons from the primary particles. As such, in this analysis, they would be incorrectly defined as primaries, affecting the accuracy of the results. However, the number of these events is expected to be relatively few, and both filters remove particles based on scattering and as such should remove these particles, resulting in a slightly underestimated specificity. Scattering with hadrons were however labeled as secondaries and all nonhadron projectiles were accounted for in this analysis.

In summary, the following metrics were used for filter comparison:

- Secondary filtering (specificity and sensitivity – simulated)
- Mean noise profile through the radiographs (simulated and experimental)
- Tomographic reconstructions of the noise (experimental)

## 3 RESULTS

The mean noise profiles of the simulated data are shown in Section 3.1.1 followed by the sensitivity and specificity plots in Section 3.1.2. Radiographs and noise profiles obtained from the experimental data are then presented along with the tomographic reconstructions and the back‐projected noise images in Sections 3.2.1 and 3.2.2, respectively.

### 3.1 Filter performance with simulated data

#### 3.1.1 Simulated radiograph noise

**FIGURE 4:**
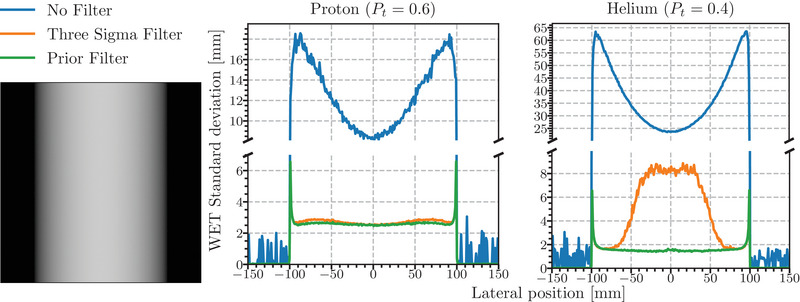
Mean noise profiles generated by averaging the standard deviation of WEPL in each pixel along the vertical direction (as viewed in the radiograph) for simulations of a proton (left) and helium (right) radiograph acquisition of a 20 cm water cylinder using different filters binned at the front‐tracker position. The threshold for the Prior Filter ($P_{t}$) was 0.6 for the proton radiograph and 0.4 for the helium radiograph. An example of the proton radiograph is shown on the left. WEPL, water‐equivalent path length.

The radiographs were generated by binning, after filtering, the particle WEPL based on the front‐tracker measurement of the entrance position with a bin‐size of 1 mm × 1 mm. The mean intrapixel noise profiles from the water cylinder simulation, obtained by averaging the standard deviation of WEPL in each pixel in the vertical direction, along with the prior filtered proton radiograph are shown in Figure 4. The *y*‐axes are broken to better resolve the difference between the two filters' profiles. The probability thresholds ($P_{t}$) for the Prior Filter were chosen to be 0.6 for the proton data and 0.4 for the helium data. For the proton data, the noise profiles filtered using the 3σ filter and Prior Filter are similar, with noise in the central region being approximately 2.5 mm of WET. The Prior Filter, however, shows a flatter noise profile remaining closer to 2.5 mm of WET than the 3σ, which rises to 2.6 mm of WET further from the center of the phantom. At the edge of the cylinder, where the most extreme range mixing occurs, both filters exhibit a sharp noise increase to approximately 7.5 mm of WET.

For the helium data, the difference between the two filters is striking; the central region of the cylinder showing the 3σ filter resulted in ≈8 mm compared to ≈2 mm for the Prior Filter. This increase is related to the higher abundance of helium ion fragments in the phantom center. These fragments skew the estimated mean and widen the standard deviation used in the filter to the extent that they are included in the 3σ envelope. A secondary reason why they are not easily filtered at the center of the cylinder is that the fragments have a higher‐than‐expected energy loss, which is closer to the primary energy loss at the thickest portion of the phantom. As in the proton data, the edges of the cylinder have sharp increases in noise, going up to 7 mm of WET.

**FIGURE 5:**
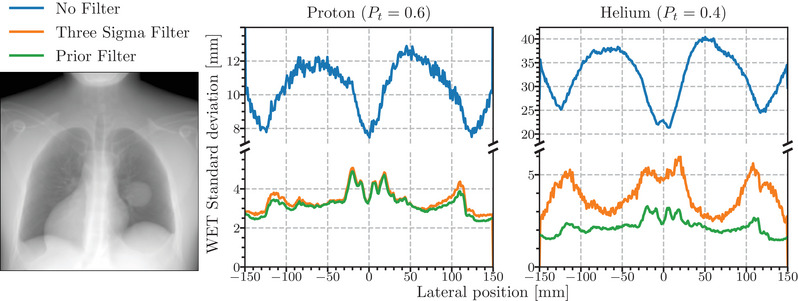
Mean noise profiles generated by averaging the standard deviation of WEPL in each pixel along the vertical direction for simulations of a proton (left) and helium (right) radiograph acquisition, of the anthropomorphic XCAT phantom, using different filters binned at the front‐tracker position. The threshold for the Prior Filter ($P_{t}$) was 0.6 for the proton radiograph and 0.4 for the helium radiograph. An example of the proton radiograph is shown on the left. WEPL, water‐equivalent path length.

Figure 5 shows mean noise profiles generated in the same way as above for the simulated radiograph acquisitions of the XCAT phantom. The radiograph was generated by binning the particle WEPL based on the front‐tracker measurement of the particle's position. The particles were binned into 1 mm × 1 mm pixels. As with the water phantom data, the noise level between the 3σ filter and the Prior Filter for the proton radiographs are similar with the Prior filter's noise profile running slightly below that of the 3σ filter. Similarly, the difference between the filters is much larger in the helium radiograph.

#### 3.1.2 Sensitivity and specificity

**FIGURE 6:**
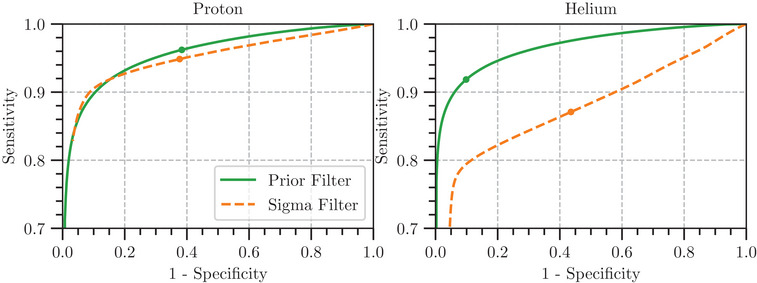
ROC curves from a simulated radiograph acquisition of a water cylinder, showing the Prior Filter (solid) and the sigma filter (dashed) for both the proton data (left) and helium data (right). The markers show the chosen threshold for reconstruction using the filters within this work. ROC, receiver operating characteristic.

Figure 6 shows the receiver operating characteristic (ROC) curves for the sigma filter and the proposed Prior Filter generated using the histories from the water cylinder simulations with protons (left) and helium ions (right). The ROC curves are created by plotting the sensitivity against 1−specificity and provide visualization of the trade‐off between the two. The thresholds chosen for image reconstruction in this work are indicated on the plots by the markers.

For the proton data it can be seen that, except for a small region corresponding to around 5σ, the Prior Filter curve runs above the sigma filter curve, indicating that a higher sensitivity is achieved for a given level of specificity. This area corresponds to a threshold of $P_{t}=0.2$. This can be considered a fairly low threshold, with many particles with a low likelihood being included within the reconstruction. For the helium data, the ROC curve of the Prior Filter remains above the sigma filter, for the range of thresholds investigated, indicating that it provides superior sensitivity for all given values of specificity. For both filters, we observe that there are no optimal points between sensitivity and specificity. Although this optimal value could be computed and applied on a case‐by‐case basis, for simplicity, a fixed value for each ion species will be used for the Prior Filter determined through preliminary testing. For the proton images, a value of 0.6 was chosen for the threshold, which it can be seen to have a similar specificity to the 3σ filter but with improved sensitivity. For the Helium images, a threshold value of 0.4 was chosen, which takes advantage of the increased sensitivity whilst maintaining high specificity.

### 3.2 Filter performance with experimental data

#### 3.2.1 Projection noise

**FIGURE 7:**
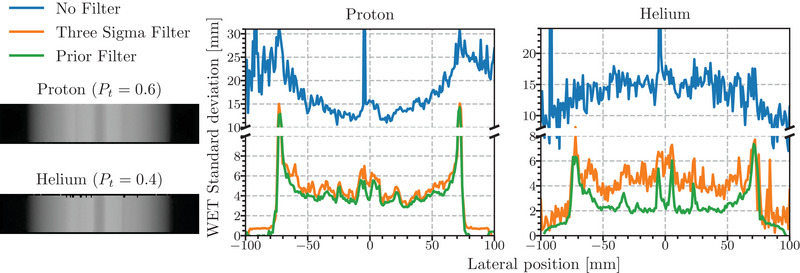
Radiographs reconstructed from both the proton (top) and helium (bottom) acquisitions of the Sensitometry module of the Catphan with binning based on the particles' entrance measurement. The Prior Filter has been applied to these images using $P_{t}$ of 0.6 and 0.4, respectively. The plots show a mean noise profile obtained from averaging the WEPL standard deviation in each pixel in the vertical direction (as viewed in the radiograph) over ± 5 pixels around the central bin for radiographs without a filter, with the 3σ filter applied and with the Prior Filter applied. WEPL, water‐equivalent path length.

Figure 7 shows the mean noise profiles from a projection (radiograph) of the CTP404 phantom acquired using the U.S. pCT prototype scanner binned based on the entrance measurement (at the front tracker plane) using a 1 mm × 1 mm bin size. The profiles were generated by averaging the WEPL standard deviation along the vertical direction in a slice ± 5 pixels around the center of the image. The results are in line with the simulated data; the proton profiles show a baseline noise performance similar in both filters (4 mm WET) in the uniform region of the phantom and rising to 5.5 mm in the section, which traverses the two air inserts. The profile from the 3σ data stays consistently above the profile of the prior filtered data, indicating more noise, with a gap up to 1 mm of WET. Similarly to the simulated data, the level of noise from both filters increases rapidly at the very edges of the object.

The helium data exhibit a larger difference. The prior filtered data have a noise value around 2.5 mm in the uniform sections of the phantom and 5 mm in the section that traverses both air inserts. The difference between the two filters in the experimental helium data is not as large when compared to the simulated data (Figure 4). This is due to the addition of the dE‐E filter in experimental data. Many of the helium ion fragments that were not removed by the 3σ filter in the simulated data have been removed by the dE‐E filter bringing its performance closer to that of the Prior Filter. For both the proton and helium profile, the difference in performance between the Prior Filter and 3σ filter is greater in the location of high‐ and low‐density inserts, indicating improved performance in heterogeneity. In the proton profile at approximately −60 mm, a sharp spike in noise for the 3σ filtered projection corresponding to the Delrin insert is not visible in the prior filtered projection. Similar differences can be seen in the Helium data (Teflon at −30 mm).

#### 3.2.2 Tomograph results

**FIGURE 8:**
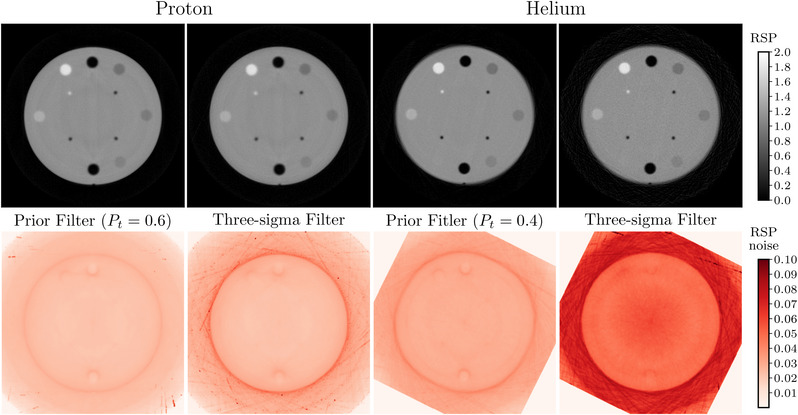
Central slice of the CTP404 Sensitometry module obtained using the tomographic reconstruction (top) and noise reconstructions (bottom) with the Prior Filter and 3σ filter applied.

The CTP404 Sensitom's RSP (top) and tomographic noise reconstructions (bottom) are shown for proton and helium in Figure 8. The noise reconstructions show similar levels of noise at the center of the phantom for the proton data, but a small benefit to using the Prior Filter can be seen towards the edges of the phantom. Again, similar to the radiograph noise, the helium data show reduced noise when using the Prior Filter across the whole phantom. All reconstructions show increased noise at the edge of the phantom and the interfaces between materials with a large difference in densities (Teflon and air inserts). These features result from range mixing caused by the scattering of particles across the boundaries of inhomogeneous regions.^40^ Table 1 contains the mean noise values within a 20 mm diameter ROI at the center of the noise reconstructions shown in Figure 8 and confirm what can be seen visually in the figure.

**TABLE 1:** Mean noise values in a 20 mm diameter ROI at the center of the noise slices in Figure 8 for the proton and helium CTs without filtering, using the 3σ filter and using the Prior Filter.

Filter	Noise [RSP]	
	pCT	HeCT
No filter	0.08	0.16
3σ filter	0.02	0.06
Prior Filter	0.02	0.03

**FIGURE 9:**
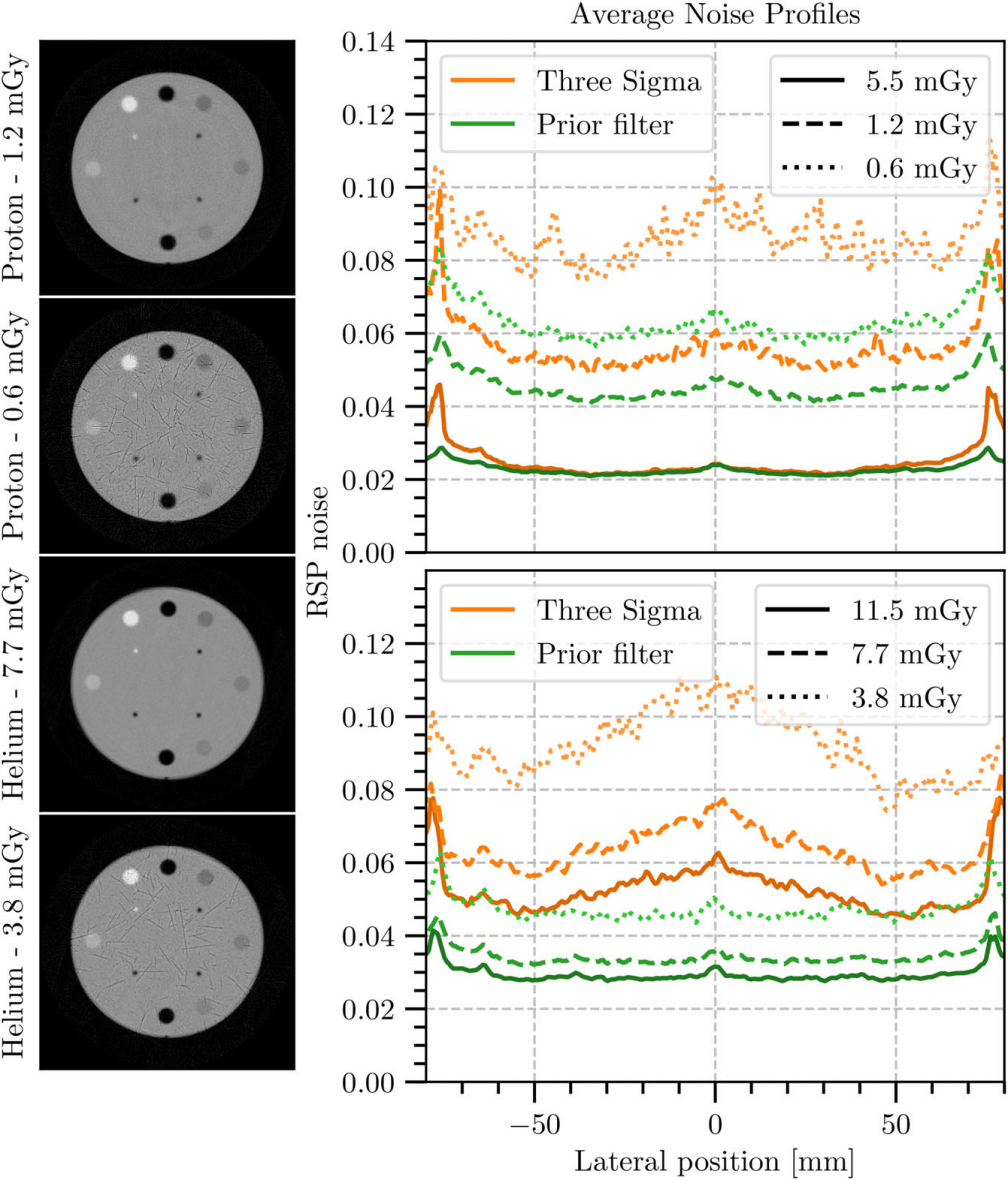
Reconstructed central slice and mean noise profiles of RSP standard deviation over ± 10 pixels from the center in the vertical direction for a range of doses showing how the 3σ filter and the Prior Filter perform with a reduced dose. The proton data are shown on top and the helium data on the bottom, with the 3σ filter in orange and the Prior Filter in green. The central slice of the reconstructions, after applying the Prior Filter, at these doses are shown on the left. The slices from the highest dose for each ion are shown in Figure 8. RSP, relative stopping power.

Following these results, we investigated further reconstructions with reduced imaging dose by removing a randomly selected portion of the incident particles before the filtering stage. These are shown in Figure 9 along with the profile from the full dose images. The initial imaging dose can be estimated by extrapolation of the simulation of the U.S. pCT collaboration prototype scanner by Piersimoni et al.^41^ To calculate the dose for this study, differences in the number of particles and the field size were accounted for with the number of detected events rather than the irradiated particles to counteract the previously mentioned inefficiencies resulting from the particle rate. For the proton CT (pCT), this was 1.8 × 10^6^ particles per projection resulting in a dose of 5.5 mGy; for the helium CT, this was 1.0 × 10^6^ particles per projection resulting in a dose of 11.5 mGy. Particles in each proton data set were reduced by factors of 4.5 and 9 to represent approximately 1.2 mGy and 0.6 mGy, respectively.

The noise profiles under a dose reduction demonstrate that the Prior Filter retains better image quality than the 3σ filter with the 0.6 mGy Prior Filter profile being closer to the 1.2 mGy profile of the 3σ filter than the 0.6 mGy 3σ filter. For the helium data, we chose smaller reduction factors (3.0 and 1.5), resulting in estimated doses of 7.7 mGy and 3.8 mGy, due to a greater proportion of primary particles being lost through nuclear interactions and fragmentation. The noise in the prior filtered data is less affected than the 3σ filtered data under these dose reductions, which demonstrates an image quality deterioration. The 3.8 mGy Prior Filter profile is below the full dose (11.5 mGy) Sigma profile for the reconstruction. This is also reflected in Table 2, which contains the mean noise in a 20 mm diameter region of interest centered on the middle slice of the noise reconstruction. Here we see the minimal impact on the noise level even with the reduction in dose of the Prior Filter when compared to the 3σ filter.

**TABLE 2:** Mean noise values in a 20 mm diameter ROI at the center of the noise slices in the same location as for the data in Table 1 for the proton and helium CTs reconstructed with low doses using the 3σ filter and using the Prior Filter.

	Noise [RSP]			
	pCT		HeCT	
Filter	1.2 mGy	0.6 mGy	7.7 mGy	3.8 mGy
3σ filter	0.06	0.09	0.07	0.11
Prior Filter	0.05	0.06	0.03	0.05

**FIGURE 10:**
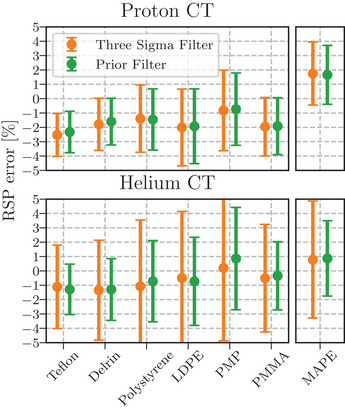
Mean and standard deviation of RSP error for the proton (top) and helium (bottom) CT using both the 3σ filter (orange) and Prior Filter (green) for each of the inserts in the sensitometry module evaluated within a cylindrical ROI 8 mm in diameter and 3 mm in height (across three slices). The mean absolute percentage error (MAPE) is indicated in the axes on the right with the mean of the standard deviations. RSP, relative stopping power.

The RSP accuracy of each of the CT reconstructions was evaluated in cylindrical ROI 8 mm in diameter, over three slices (3 mm cylinder height) within each insert. The RSP percentage error of each insert is given in Figure 10 with the mean absolute percentage error (MAPE) for each reconstruction. The RSP is underestimated for all inserts, except for the PMP insert. For the proton images, the MAPE were 1.66% and 1.75% for the Prior filtered and 3σ filtered tomographs, respectively, and 0.87% versus 0.79% for helium. Additionally, the standard deviation of RSP error within each insert is reduced for the Prior Filter indicating a reduced noise.

## 4 DISCUSSION

The results shown in this work emphasize the conclusions that filtering in ion imaging is essential for high image quality.^3^, ^13^, ^15^ The 3σ filter is currently in common use in particle imaging research. This work has shown that our Prior Filter returns equivalent or slightly superior performance in proton imaging in terms of image noise. Additionally, it has been shown to have improved performance over the 3σ filter when applied to helium imaging as a result of the greater ability to filter secondary fragments, which can be determined from the sensitivity and specificity plots (Figure 6). Although both filters depend on estimating the parameters of normal distributions, the Prior Filter estimates the expected position and energy loss of individual particles rather than characterizing an ensemble of particles within a single bin. Khellaf et al.^10^ have previously discussed the implication of heterogeneous objects and the resulting multiple energy peaks on sigma filtering. In the same work, they show that the assumptions of the MLP are inaccurate at steep lateral density gradients. By relying on the MLP to make predictions of a particle's exit parameters, the accuracy of the prediction is tied to the accuracy of the path estimate. In this work, it has been shown that the path estimates used in the Prior Filter are sufficiently accurate to provide better filtering for inhomogeneous materials; see the XCAT phantom simulations in Figure 5. The accuracy of the filter would only improve with more accurate path estimates.

The MAPE of the RSP inserts were higher than previously reported, see Giacommetti et al.^34^ for proton imaging and Volz et al.^15^ for helium imaging. The systematic underestimate of RSP for reconstructions using both filters indicates a calibration error that is not picked up by the Prior Filter. Indeed, a recent publication by Dickmann et al.,^42^ using the same prototype scanner, found a systematic shift in RSP values of −1.3%. Additionally, pCT reconstructions with an iterative reconstruction algorithm,^32^ reported higher RSP accuracy (<1%). However, the reconstruction algorithm in this study was selected for its ability to generate pixel noise tomographs for the filter evaluation, with the main goal being the comparison of filter methods rather than the precision of the reconstruction.

Aside from performance, there are some further advantages to the use of the filter proposed in this work. The filter itself is based on the physical principles of particle transport in matter, as opposed to the data‐based estimates underlying the 3σ filter. Additionally, the acquisition data are filtered on a particle‐by‐particle basis, that is, each particle gets its own estimation of WEPL and scattering angle (indirectly through the position estimate), while the 3σ filter assigns an estimate to each bin, requiring sufficient statistics. The Prior Filter performance should be independent of imaging dose, so long as we have good prior information. This is supported by the results presented in Figure 9. Conversely, the 3σ filter makes the assumption that the number of particles in each image bin is adequate so that the moments of its distribution are related to the individual particle characteristics. At low dose, this assumption drops, and the 3σ filter performance suffers (see Figure 9).

An interesting feature of this filter is that it is easily adjustable through the use of different probability thresholds ($P_{t}$). The sigma filter is also adjustable but is dependent on estimates from the distribution per pixel rather than individual particle estimates, which can be vulnerable to heterogeneity. Though only the true particle path will eliminate this effect, the benefit of the Prior Filter can be highlighted by thinking of a pixel at a high gradient edge. The distribution in said pixel will have two peaks. The 3σ filter would not reflect well either distribution, while the Prior Filter acts on each distribution individually.

Although in this work a fixed value was used, it would be possible to optimize this value based on an image quality metric to tailor the performance to the priorities of the use case. Deviations from expected position and WEPL are linked, however, the three variables (measured WEPL probability, measured Y probability, and measured Z probability) might be related to various nonelectromagnetic interactions, and have three separate optimal values, providing further opportunity to tune performance. For example, in helium imaging where particle fragments are more abundant and scattering reduced when compared to proton imaging, emphasis can be placed on the WEPL probability should there be a need to conserve particles. The optimal threshold under different use cases is a focus of further study.

By reducing the noise in the global signal, our filter increases the inherent information carried by each particle and fewer particles are needed to reconstruct an image with an adequate signal‐to‐noise ratio. This possibility is demonstrated in the low‐dose imaging Figure 9, which may contribute to realizing ultra‐low‐dose particle imaging. The 3σ filter noise increases faster as the dose is reduced showing that the performance of the 3σ filter is being degraded. A possible method of improving the 3σ filter's performance with fewer particles is to increase the bin size to ensure that there are enough particles per bin to calculate the appropriate parameters. To test this, the dimensions of each bin were tripled (nine‐fold area increase) for the 0.6 mGy reconstruction before filtering and reconstructing the data again. This reconstruction has a mean RSP noise of 0.07 in the same 20 mm diameter region explored in Table 2 with noise level across the phantom between the 1.2 and 0.6 mGy 3σ reconstructions, which can be seen in Appendix C. Thus, the noise can be reduced with a larger bin size; however, it is worth noting that this phantom is mostly homogeneous and for less homogeneous phantoms, the larger bin size increases the likelihood that a given bin will contain a heterogeneity possibly affecting the filter's efficiency.^10^ Although the images reconstructed using the 0.6 mGy data contain artifacts from voxels that protons have not passed through, techniques such as sinogram interpolation^43^ can be used to reduce the impact of the artifacts. Other image improvement methods, such as Gaussian deblurring,^44^ are affected by the noise and would also benefit from the proposed filter.

Still, there are some disadvantages to the Prior Filter when compared to the 3σ filter. The requirement of prior 3D information to filter the data limits its applicability to filtering radiographs unless a previous image or data from other modalities such as single‐energy CT or dual‐energy CT are used to provide the prior information. This comes with additional complications of registration and RSP uncertainties. In the case of particle CT, an initial reconstruction can be used in order to filter the data before a final reconstruction is performed on the filtered data, as shown in this study. This does again require an increase in total reconstruction time when including the initial reconstruction. However, the value of using a prior reconstruction for hull detection has previously been demonstrated^25^ and as such, this step is not expected to greatly impact the overall reconstruction time of quality particle CT images. Even in this case, however, the number of computations required is greater than the relatively simple 3σ filter due to the need to estimate the path for each particle in the filtering step. No estimates for differences in time are given in this work as neither filter was implemented optimally and to do so would not be representative of achievable speeds for either filter. One advantage of single‐event imaging is that the independence of each measurement allows the data processing to be highly parallelizable.

## 5 CONCLUSIONS

A novel filtering technique based on an analytical model of electromagnetic interactions has been proposed and evaluated against distribution‐based filtering techniques for both proton and helium imaging. The filter uses the models of multiple Coulomb scattering and particle energy loss to predict the likelihood that a measured data point had not undergone nuclear interactions or large angle scattering, that is, had interacted through the primary electromagnetic model only. The performance of the filter, applied to both simulated and experimental data, was found to be comparable to the commonly used 3σ cut when applied to proton imaging. The same comparison showed the proposed filter reduced noise in both helium radiographs and tomographs. Investigation into the sensitivity and specificity of the filters showed the improved sensitivity over a range of sigma cuts although, in the proton images especially, this came at reduced specificity. The performance of the filter was evaluated for lower dose imaging and found to be much more consistent than the 3σ filter. This results from an appealing feature of the proposed filter; that each particle is filtered independently of the others. Other potential advantages of using such a filter are its basis in the physical processes being investigated and the potential to optimize the probability threshold based on the use case. This does, however, come at the cost of increased complexity with the requirement of prior knowledge and more demanding computation.

## CONFLICT OF INTEREST STATEMENT

The authors declare no conflicts of interest.

## Figures and Tables

**FIGURE A1:**
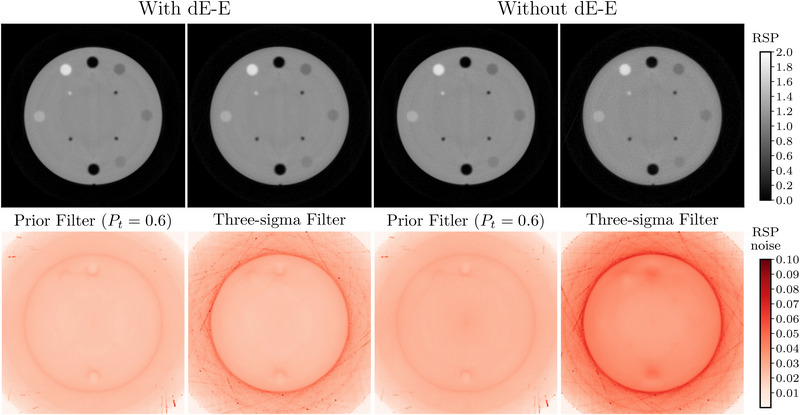
Central slice of the CTP404 Sensitometry module obtained using the tomographic reconstruction (top) and noise reconstructions (bottom) with the Prior Filter and 3σ filter applied without the dE‐E filter for the proton tomographic data in this work.

**FIGURE A2:**
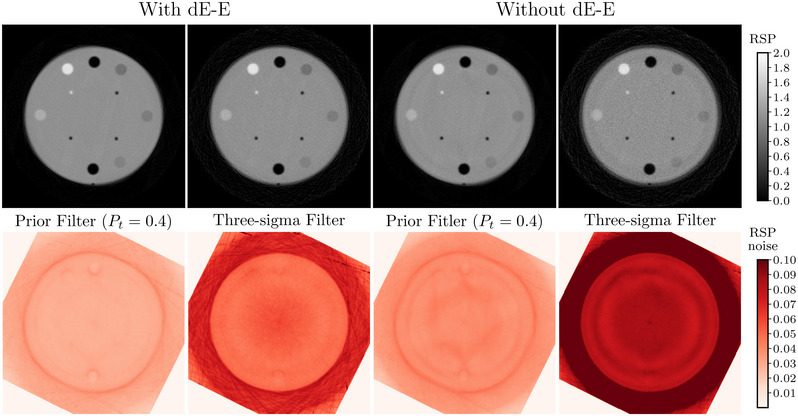
Central slice of the CTP404 Sensitometry module obtained using the tomographic reconstruction (top) and noise reconstructions (bottom) with the Prior Filter and 3σ filter applied without the dE‐E filter for the helium tomographic data in this work.

**FIGURE A3:**
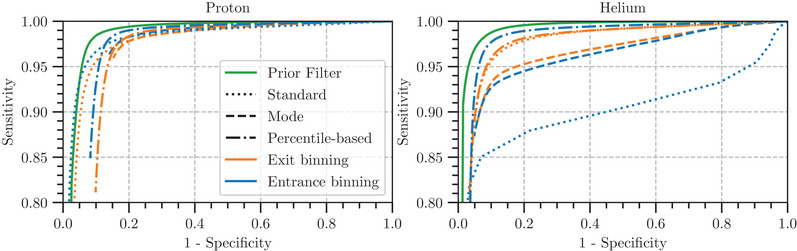
ROC curves from a simulated radiograph acquisition of an anatomical XCAT phantom, showing the Prior Filter (solid) and the standard sigma filter (dots), the mode‐based sigma filter (dashes) and the percentile‐based sigma filter (dash‐dot) with both entrance (blue) and exit (orange) binning for both the proton data (left) and helium data (right). ROC, receiver operating characteristic.

**FIGURE A4:**
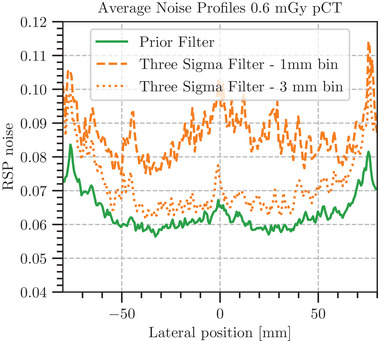
Mean noise profiles of RSP standard deviation for proton CT reconstructions of the CTP404 sensitometry phantom using particles corresponding to a dose of 0.6 mGy as shown in Figure 9 for the Prior Filter and 3σ filter including a 3σ filtered profile that has been filtered with a bin size of 3 mm bin size.

## APPENDIX A FILTER PERFORMANCE EXCLUDING DE‐E FILTER

For completeness and to show that the results of this work hold when separated from the dE‐E filter, Figures A1 and A2 show the results of the tomographic noise reconstructions with and without the application of the dE‐E filter for proton and helium ions, respectively. These figures show that in both cases, the reconstructions without the dE‐E filter applied, contain more noise as a result of the inclusion of more secondary particles. As expected, this is especially the case for the helium ions for which this filter is designed, but a visible effect is seen for the proton images. This is backed up by the values in Table A1, which show the mean noise within a 20 mm diameter ROI at the center of the noise images without the dE‐E filter applied.

**TABLE A1:** Mean noise values in a 20 mm diameter ROI at the center of the noise slices in Figures A1 and A2 for the proton and helium CTs without the dE‐E filter applied.

	Noise [RSP]	
Filter	pCT	HeCT
3σ filter	0.03	0.06
Prior Filter	0.03	0.03

Results are given for the reconstruction with using the 3σ filter and using the Prior Filter.

## APPENDIX B PERFORMANCE OF THE SIGMA FILTER WITH DIFFERENT IMPLEMENTATIONS

Due to the various implementations of the sigma filter in the literature, this appendix includes a comparison of different implementations to show that the Prior Filter outperformed them all in discriminating secondary events. For comparison, ROC curves, as in Section 3.1.2, were calculated based on a simulation of a radiograph acquisition of the XCAT phantom as described in Section 2.2 between 0σ and 10σ. The following implementations of the sigma filter were compared:

- Standard – All particles are binned by their position measurement and all particles are used in the calculation of the bin mean and standard deviation used to make the sigma cuts.^3^, ^9^
- Mode – All particles are binned by their position measurement; however, only particles within 30% of the bin mode are used in the calculation of mean and standard deviation used to make the sigma cuts.^13^
- Percentile‐based – All particles are binned by their position measurement. However, rather than the mean, the median was used and the standard deviation is calculated by taking the difference between the median and the 30.85th percentile (corresponding to 0.5σ) and multiplying it by 2.^14^

For each method, 1 mm × 1 mm bins were used, and each method was implemented with binning based on both the measured front and rear tracker positions. For the proton data, similar performance is seen for all methods with the standard method showing a preference towards specificity and the percentile‐based binning showing slightly better sensitivity than the other methods. Little difference is seen between the front and rear tracker binning. In the helium data, there is a greater difference in performance between the different methods and here the benefits of removing the tails of the distribution can be seen by the poor performance of the standard binning method compared to the two that calculate the distribution parameters on a smaller subset of the binned data. In both proton and helium ions, the Prior Filter shows superior performance to all methods (Figure A3).

## APPENDIX C THREE SIGMA FILTER WITH AN INCREASED BIN SIZE

Figure A4 shows the 0.6 mGy noise profiles from the reconstruction of the CTP404 sensitometry phantom as described in Section 3.2.2. The profiles shown include the data reconstructed with the Prior Filter ($P_{t}=0.6$) and the 3σ filter applied from Figure 9. Additionally, a profile from a reconstruction that was filtered using the 3σ filter with an increased bin size, of 3 mm, to demonstrate the effect of increasing the bin size to maintain the same number of particles per bin when using this filter. The increased bin size does reduce the overall noise in the phantom, but not to the point where it is equal to the Prior Filter. Additionally, this phantom is mostly uniform which reduces the negative impacts of increasing the bin size such as splitting the distribution as described in Khellaf et al.^10^ Some of the effect can be seen in the negative side of the *x* axis, where the noise increases sharply, due to the higher density inserts on this side.
